# Blast loads and variability on cylindrical shells under different charge orientations

**DOI:** 10.1038/s41598-023-30785-8

**Published:** 2023-04-25

**Authors:** Fei Yin, Xudong Zhi, Feng Fan, Wuchen Wei, Dianshuang Zheng

**Affiliations:** 1 Key Lab of Structures Dynamic Behaviour and Control of the Ministry of Education, Harbin Institute of Technology, Harbin, 150090 China; 2 Key Lab of Smart Prevention and Mitigation of Civil Engineering Disasters of the Ministry of Industry and Information Technology, Harbin Institute of Technology, Harbin, 150090 China; 3 Norinco Group, Aviation Ammunition Institute, Harbin, 150030 China

**Keywords:** Mechanical engineering, Civil engineering

## Abstract

Cylindrical shells are widely used in public buildings and military protection fields, and it has a high risk of terrorist attacks and military attacks, it is of great social benefit to carry out the anti-blast design of cylindrical shells, which needs to consider building shape and the shape of blast waves. In this paper, cylindrical charges in five directions were detonated on the outer ground of the scaled cylindrical shell, blast loads of the cylindrical shell were measured and blast waves were photographed. The variation of blast load is analyzed by combining the test and simulation results, the difference in peak overpressure of the blast waves on the end face between five orientations is nearly twice. The blast loads in the axial direction of cylindrical charges have a secondary peak phenomenon, and the blast loads between the axial direction and radial direction of cylindrical charges change abruptly at a specific angle. The experimental and simulation methods provide a reference for establishing a blast load database of typical buildings.

## Introduction

**Figure 1:**
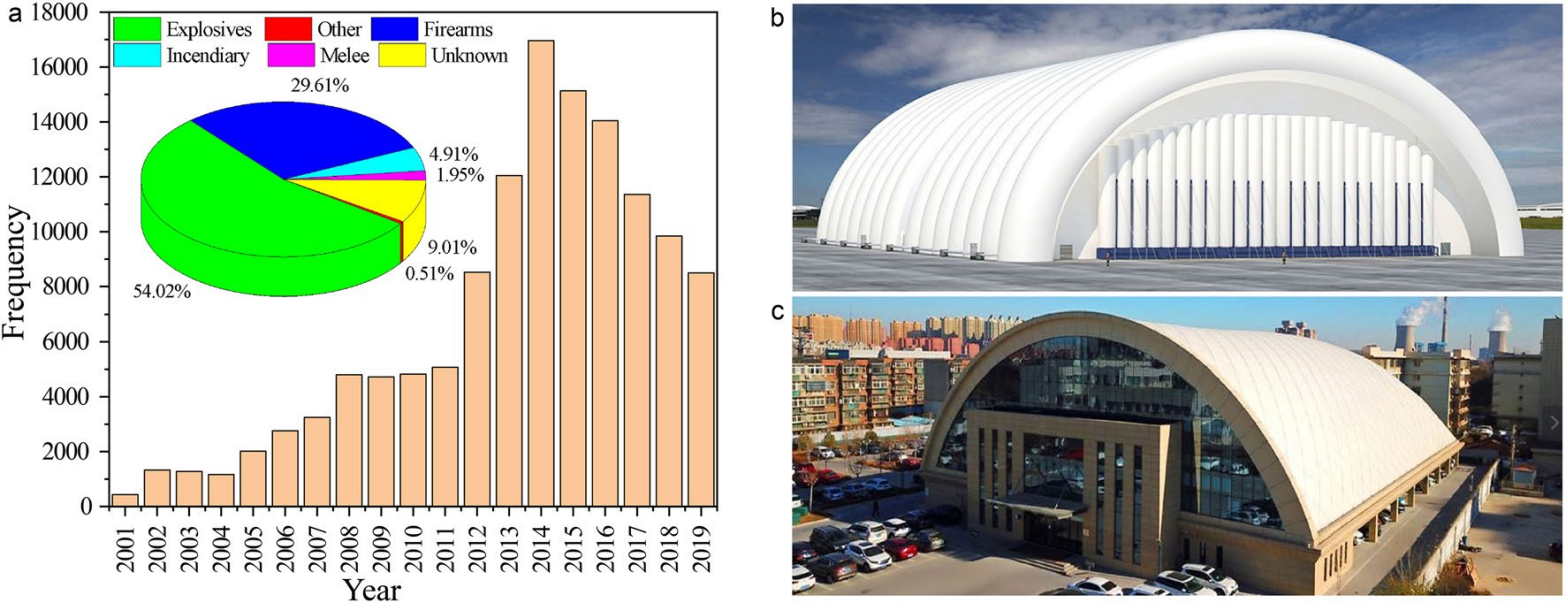
research motivation. (**a**) Global distribution of terrorist attacks^3^. (**b**) Mobile hangar. (**c**) Inflatable stadium.

Accidental blasts and terrorist attacks have occurred frequently in recent years, with the number of terrorist attacks rising to more than 10,000 times per year, more than half of which are bombings (Fig. 1a). Cylindrical shells (Fig. 1b,c) are widely used in military fields and important infrastructure, which are at greater risk of military attacks and terrorist attacks, it has great social benefit to improve the blast resistance of cylindrical shells^1,2^.

The anti-blast design of buildings should first obtain blast loads of buildings, in the past, scholars pay more attention to spherical charges^4,5^ and the blast loads of flat plates^6,7^, the widely used empirical formulas were proposed by Kingery and Bulmash^8^, which were also written into design manuals^9^ and numerical programs^10^, however, these empirical formulas differ significantly in the near-field blasts, which is often attributed to the variability of blast waves due to the physical mechanism for this phenomenon is not clearly understood^11,12^. However, the representation method of scaled distance in the empirical formula defaults to the blast waves as spherical waves, For the real scene of bomb attacks, the blast waves in near-field blasts which poses a great threat to buildings are usually non-spherical^13^, owing to the shape of charges, even spherical charges are difficult to detonate symmetrically in actual tests^14^. The load variability caused by ignoring the directionality of blast waves is not clear.

The geometric shape of buildings also has an important impact on blast loads^15–18^, there is no detailed test data on blast loads of cylindrical shells^19^, and the test data in near-field blasts is very scarce, owing to traditional gauges can not withstand such a large pressure and high temperature of the flame^20^, and it also limited by the number of gauges. Therefore, the complex propagation of blast waves was recorded by high-speed photography techniques to facilitate the analysis of blast loads, and the accuracy of the numerical model is fully verified.

In this paper, the influence of detailed factors such as charge shape and detonation configuration^13,21–23^ was also considered, and the object of blast loads was selected as the cylindrical shell with less test data^24^. Considering that the response speed of the building structure is slow and the coupling effect with the blast wave is not large, the rigid model is used to test blast loads^25–27^. The static images showing blast waves intuitively through pixel processing^28^, and detailed test data of blast loads on the cylindrical shell were obtained. The influence of charge orientation and the formation process of blast loads were analyzed by tests and simulations.

## Results

### Test arrangement

**Figure 2:**
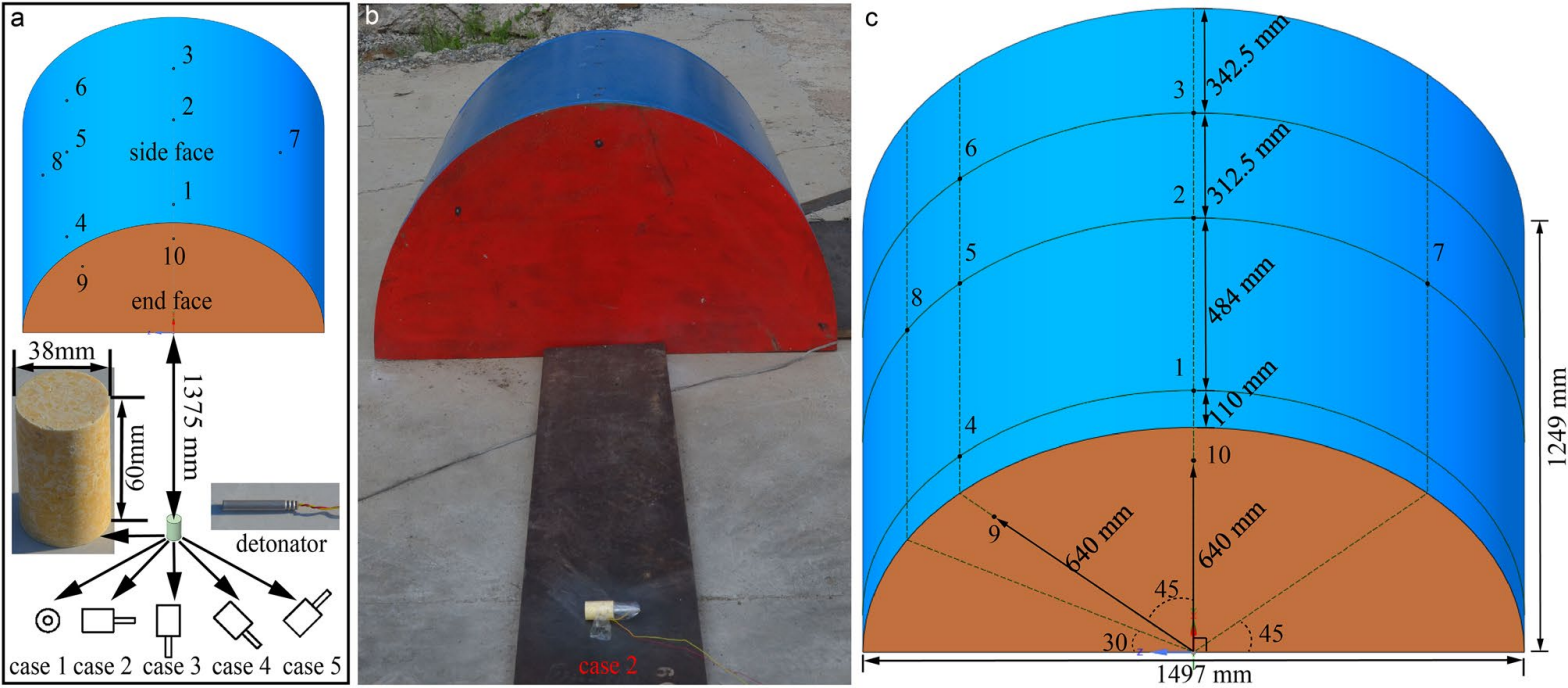
Test arrangement. (**a**) Location of charges, detonators and cylindrical shells. (**b**) Test 3 of Case 2. (**c**) Location of 10 gauges.

The charge mass is 110 ± 1 g (Fig. 2a), one end of the cylindrical charge containing the detonator is the tail, and the other end is the head. Each case is tested twice to ensure the reliability of the test results. The cylindrical shell is made of Q 355 (Yield strength < 355 MPa) steel with a thickness of 30 mm, and the steel plate with the thickness of 30 mm is placed under the charges to ensure the same initial conditions, a ground wire is arranged between the steel plate and the cylindrical shell to shield the electromagnetic signal during detonation, and cables of gauges are protected by covering triangle steels (Fig. 2b). A total of ten gauges are arranged on the shell surface (Fig. 2c).

### Overpressure-time histories

**Figure 3:**
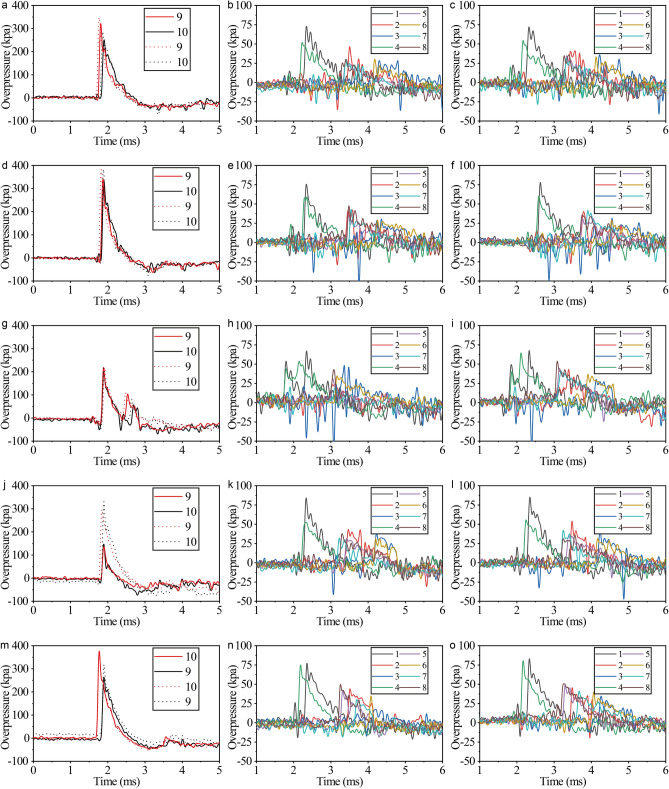
Overpressure-time histories. (**a-c**) Case 1 (adapted from Ref.^15^). (**d-f**) Case 2. (**g-i**) Case 3. (**j-l**) Case 4. (**m–o**) Case 5.

The overpressure histories (Fig. 3a-o) are low-pass filtered by Butterworth method (IIR) in ORIGIN software^29,30^, the negative pressure of some gauges in Case 2 and Case 3 could not return to atmospheric pressure for a long time, this is the thermal transients^31^ of gauges caused by the fireball. Overpressure-time histories for the repeated tests showed good consistency, there are significant differences in the test results of different charge orientations compared with repeated tests, including the arrival order of blast waves, peak overpressure and curve shape.

### High-speed camera results

**Figure 4:**
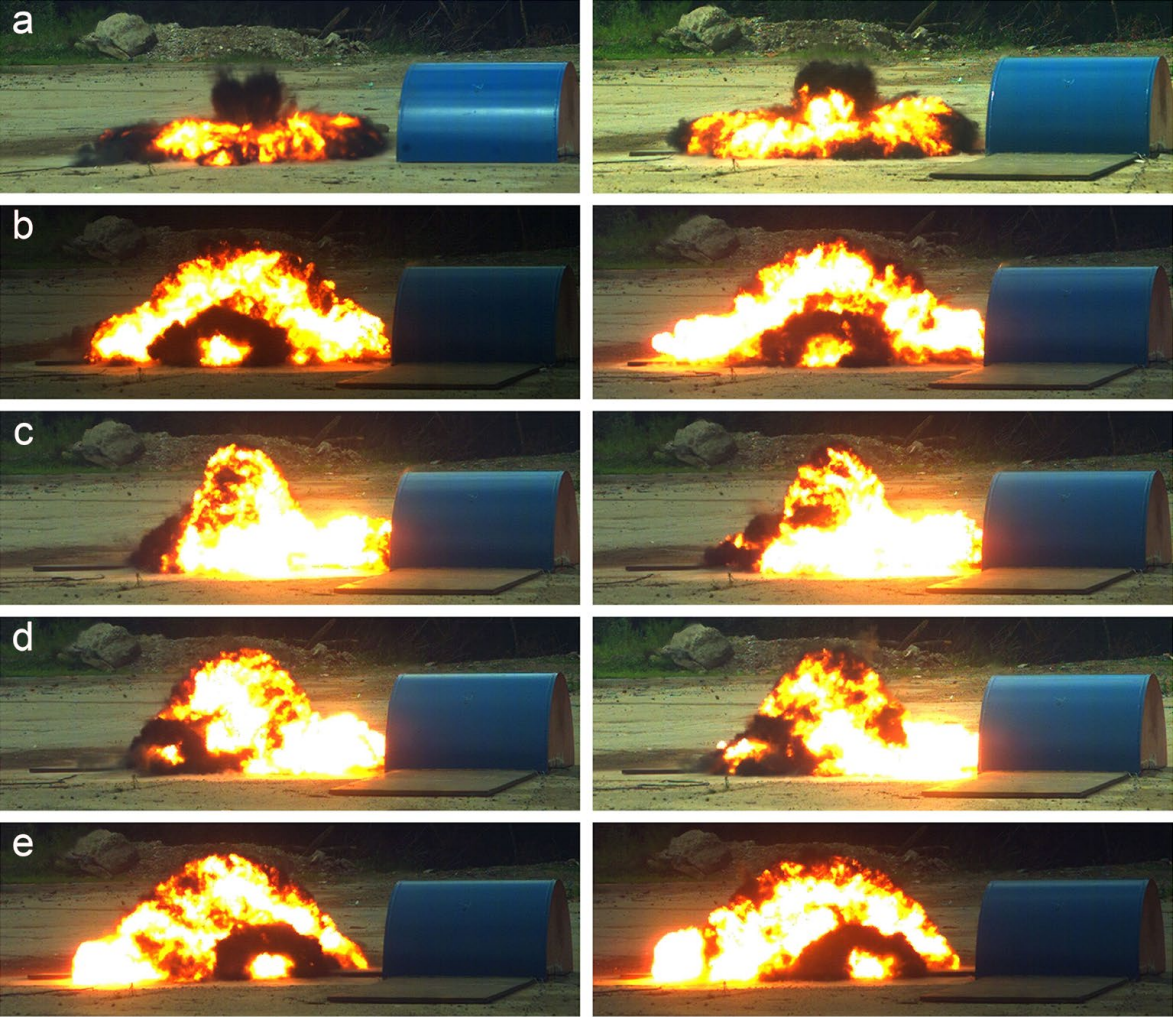
Detonation products of repeated tests at 2 ms. (**a**) Case 1. (**b**) Case 2. (**c**) Case 3. (**d**) Case 4. (**e**) Case 5.

The fireballs in repeated tests are consistent while the fireballs under different charge orientations are significantly different (Fig. 4a–e). The fireballs in Case 1 (Fig. 4a) spread rapidly toward the ground and bounces back toward the middle. From Case 2 to Case 5, the fireballs first expand rapidly with blast waves towards the axial direction of cylindrical charges away from detonators (Fig. 4c), then form black smoke at the location of detonators, the fireball finally slows down and spreads around. The fireballs reflect the shape of blast waves^32^ and change the state of air^33^ in the near field, they are non-spherical and have obvious directivity in all cases.

**Figure 5:**
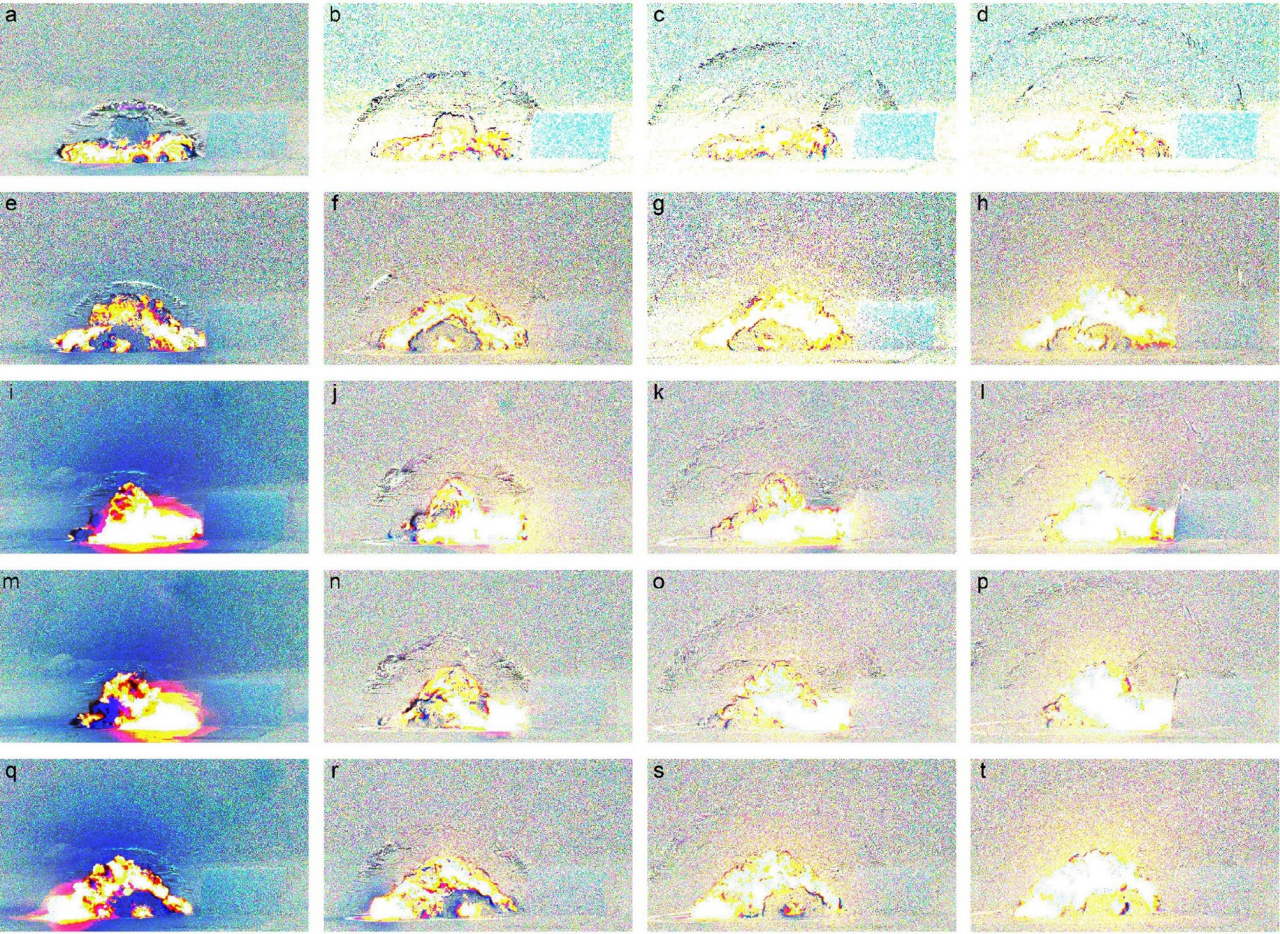
The blast waves at 1 ms, 2 ms, 3 ms, and 4 ms. (**a-d**) Case 1. (**e–h**) Case 2. (**i-l**) Case 3. (**m-p**) Case 4. (**q-t**) Case 5.

Static images of blast waves (Fig. 5) are obtained by subtracting the pixels of the previous image, increasing brightness, and enhancing the contrast, and the blast waves are more obvious than the original photo (Fig. 4). After the primary shock wave separating from the fireballs, it rapidly evolves into an ellipsoid wave (Fig. 5a), and then collides with the end face and forms a reflected wave (Fig. 5b), finally, the reflected wave collides with the secondary shock wave (Fig. 5c) to form a high-pressure area and bounce back^15,34,35^, the two waves seem to remain in their original trajectories without interference (Fig. 5d).

Whether cylindrical charges are detonated in the air^36,37^ or on the ground, the blast waves expand faster along the radial direction of cylindrical charges (Fig. 5e,f) than along the axial direction (Fig. 5i,j), therefore, the blast loads are relatively large (Fig. 3) when the radial plane of cylindrical charges (Slenderness ratio L/D = 1.58) passes through structures. Compared with the images in Case 4 (Fig. 5m–p) and Case 5 (Fig. 5q–t), the position of detonators has less effect on blast waves than on fireballs.

## Discussion

**Figure 6:**
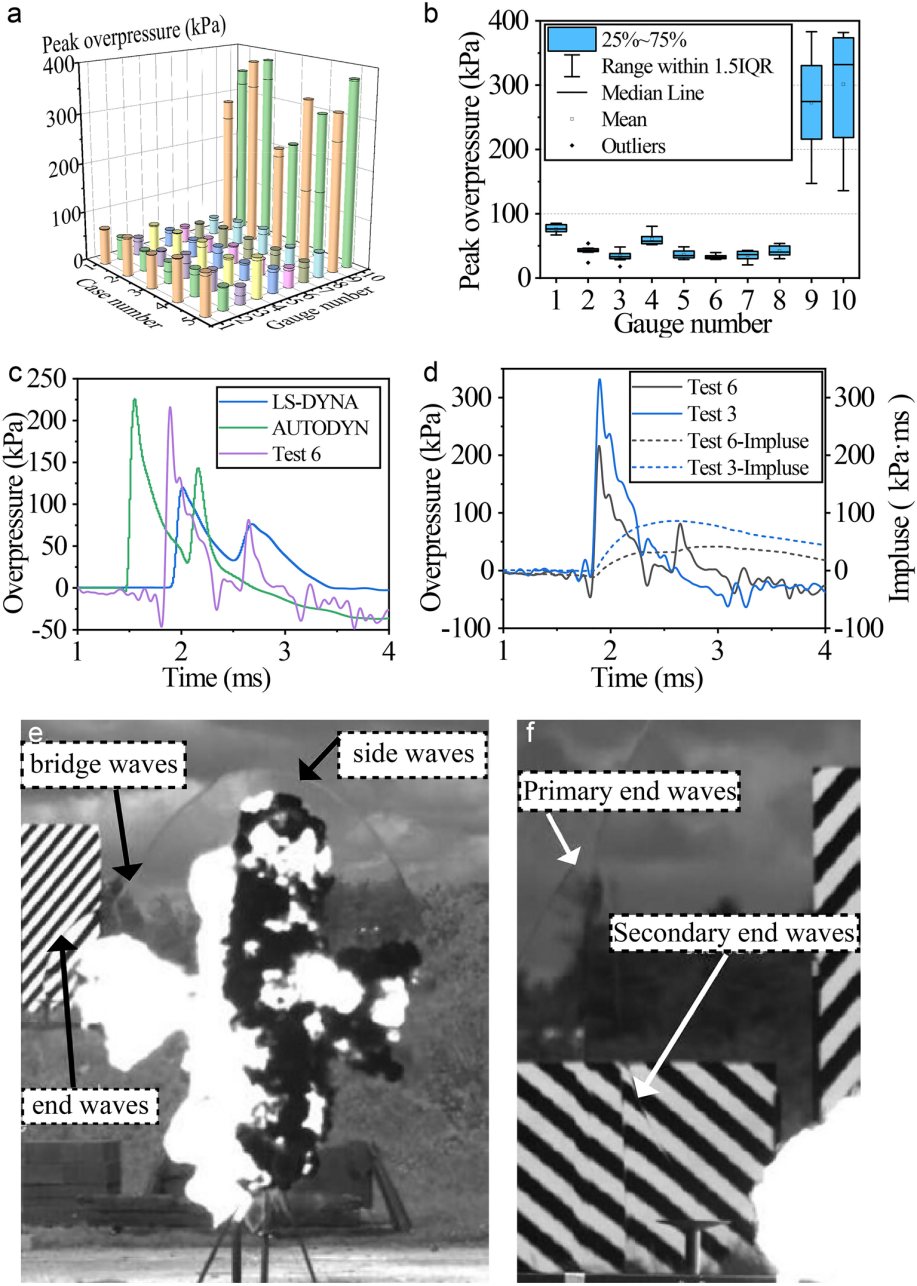
Variability analysis of blast loads. (**a**) Distribution of peak overpressures. (**b**) Statistical variability of peak overpressure. (**c**) Test and simulation results at Gauge 10. (**d**) Comparison of Case 3 and Case 2. (**e**) The detonation products and blast waves of cylindrical charges (0.459 kg at 3.6 ms) under air blasts (adapted from Ref.^37^). (**f**) Primary end waves and secondary end waves.

The peak overpressure on the end face is larger than that on the side face owing to charges being close to the end face, and the peak overpressure at the edge of cylindrical shells attenuates by about 75% (Fig. 6a). If the charge orientation is considered as an uncertain factor in the actual situation, the variability of peak overpressures at Gauge 9 and Gauge 10 is very large, both the mean and the variability of peak overpressure gradually decrease as the blast wave propagates, the peak overpressure on the side face has small signal-to-noise ratio and outliers (Fig. 6b).

The overpressure time histories in Case 3 (Fig. 3g–i) has two peaks, The appearance of two peaks may be influenced by factors such as flame interference, multiple reflections of waves^38–40^, edge clearing effects, charge shape^37^ and detonation configuration, judging from the interval time between the two peaks, the most likely cause is the extension wave of bridge waves behind end waves of cylindrical charges (also known as secondary end waves^36^). Simulations were carried out in LS-DYNA software^41,42^ and AUTODYN software^20,43^ respectively (described in the next section), the two peaks of the simulation results in LS-DYNA are smaller than test results, and it was found that the test and simulation achieved consistent results when the charge was deflected upwards by 9.4 degrees in AUTODYN (Fig. 6c). Compared with other cases, the peak overpressures in Case 3 is significantly smaller (Fig. 6a) and the maximum impulse is also significantly reduced (Fig. 6d).

The results of repeated tests in Case 4 are quite different (Fig. 3j), considering that the overpressure of Gauge 9 and Gauge 10 decreases at the same time, and the other gauges have good repeatability, which precludes the failure of some gauges or the incomplete detonation of charges. In addition, it should not be caused by the interference of gauges by fireballs, after all, the fireballs in the two repeated tests were similar (Fig. 4d). The charge orientation in Case 4 is a compromise between Case 2 and Case 3, coincidentally, the results of Test 7 is similar to that of Case 3, while the result of Test 8 (Similar to the results in AUTODYN) is similar to that of Case 2, therefore, it is possible that the charge or detonator in Test 7 has an angle deviation, this also indicates that the load difference between Case 2 and Case 3 is not a slow transition, but a sudden change (The blast waves of cylindrical charges are more complex in this direction and the peak pressure tends to change abruptly^44^) occurs in the charge orientation of Case 4.

The fireball (Fig. 4a) and blast waves (Fig. 5a) in Case 1 is similar to half of the fireball in the air blasts of cylindrical charges (Fig. 6e), owing to the mirror reflection of the ground. There are obvious junction and stratification between primary end waves and secondary end waves (Fig. 6f).

**Figure 7:**
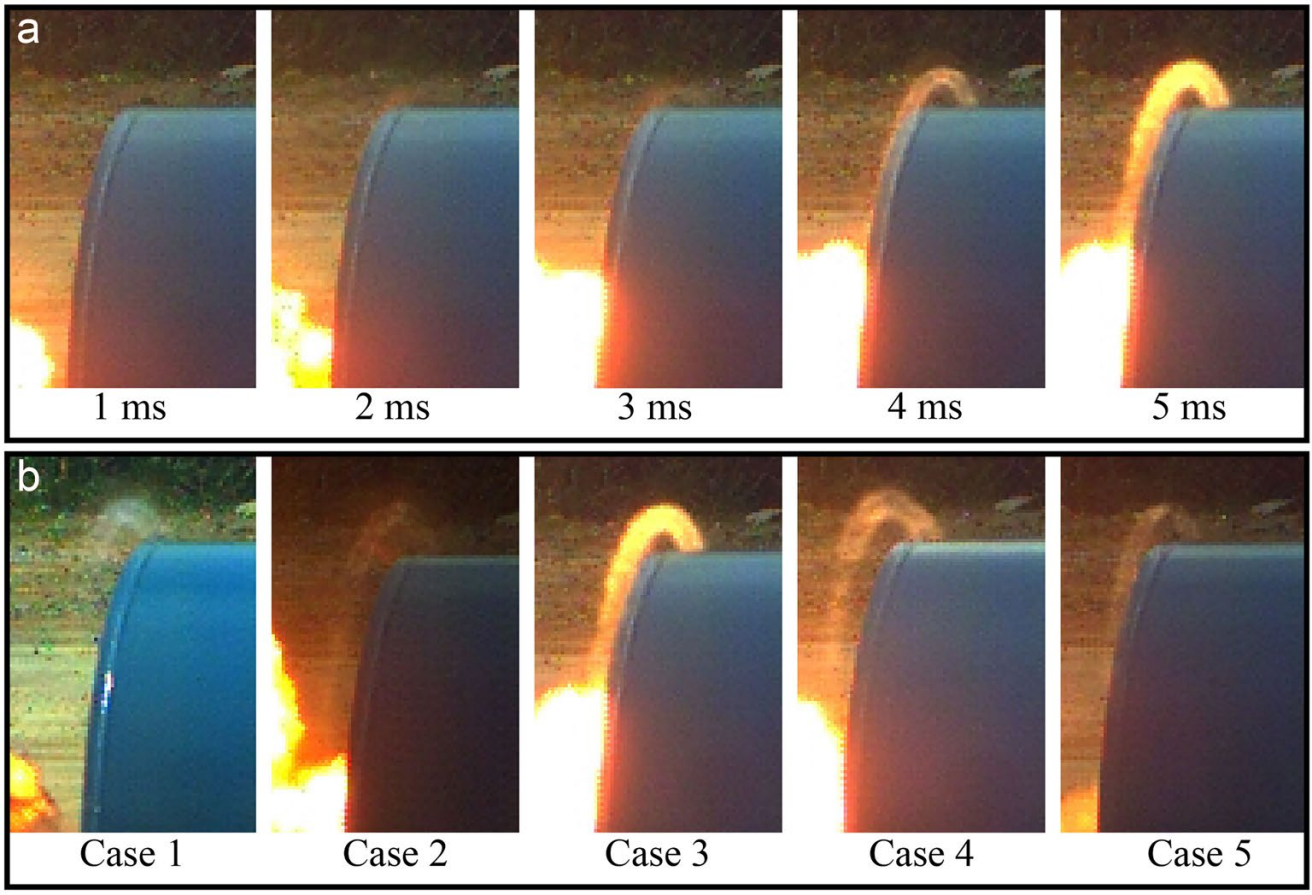
Vortex rings. (**a**) Vortex rings of Case 3. (**b**) Vortex rings at 5 ms.

The detonation gas rotates (Fig. 7a) at high speed near the edge of cylindrical shells and burns again, forming stable vortex rings (Fig. 7b) and propagating away from the end face. The propagation of vortex rings conflicts with previous research assumptions^45,46^, visualized images help understand clearing effects at model edges (this flow phenomenon conflicts with the assumptions of the Hudson method^47^), both AUTODYN or LS-DYNA have difficulty simulating this complex flow phenomenon.

## Methods

### Test methods

The interest in blast-resistant design is that structures cannot be completely destroyed by blast waves, the reason for using the rigid shell to test blast loads is that the velocity of blast waves is much higher than the response speed of structures, and the deformation of structures will hardly affect blast waves. A pre-blast was performed before gauges were installed, and the cylindrical shell was found to be heavy enough not to move. The tests were carried out on a remote mountain with no radio signals around, the cylindrical shell and bunkers were installed by a magnetic crane and 2 diesel generators provided electricity for the equipment.

**Figure 8:**
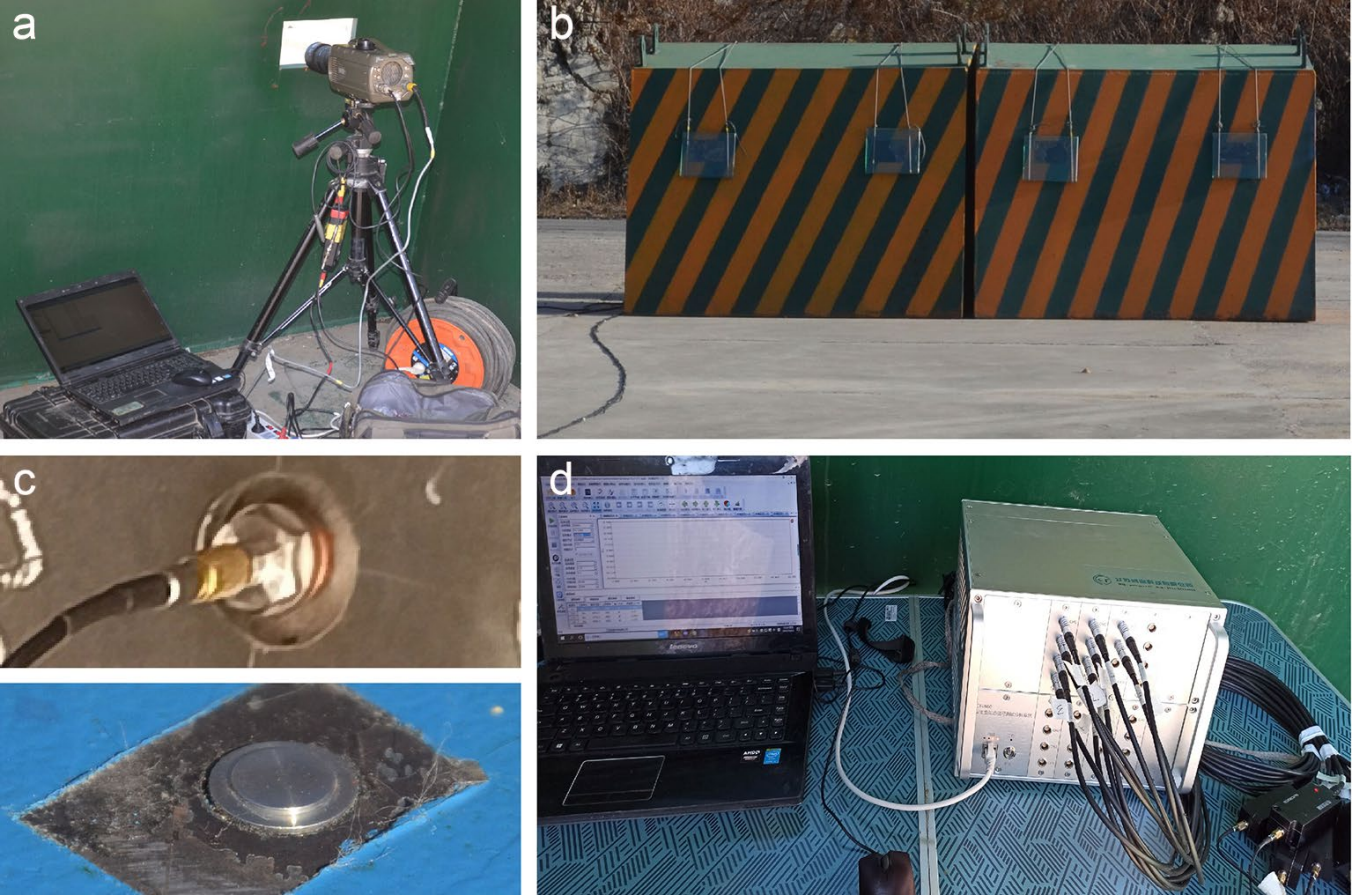
Test methods. (**a**) High-speed camera. (**b**) Bunkers with glass windows. (**c**) Gauges inside and outside the shell. (**d**) Charge debugger, dynamic data acquisition, and processing system.

The high-speed camera shot through bulletproof glass (Fig. 8a). Testers and instruments are in a bunker 20 m from the blast site (Fig. 8b). The gauges were screwed out by internal thread and spacers of different thicknesses were added to ensure that the surface is flush with the shell surface (Fig. 8c). The piezoelectric signals are transmitted through 30 m graphite-shielded cables covered with protective angle steel, and they are connected to the acquisition instrument through charge debuggers (Fig. 8d). Finally, it is stored and processed by the matching dynamic signal processing system on the computer (Fig. 8d), the sensitivity of gauges was calibrated before tests and input to the processing system, the sampling frequency of signals is set to 200 kHz (filtering after recording the original signals) and the shooting frequency is 10,000 fps. The atmospheric conditions of field tests were simply recorded, the temperature is 35 ± 7 °C, the humidity is 46 ± 10%, and the air pressure is 95.5 kPa^48^.

### Simulation methods

**Figure 9:**
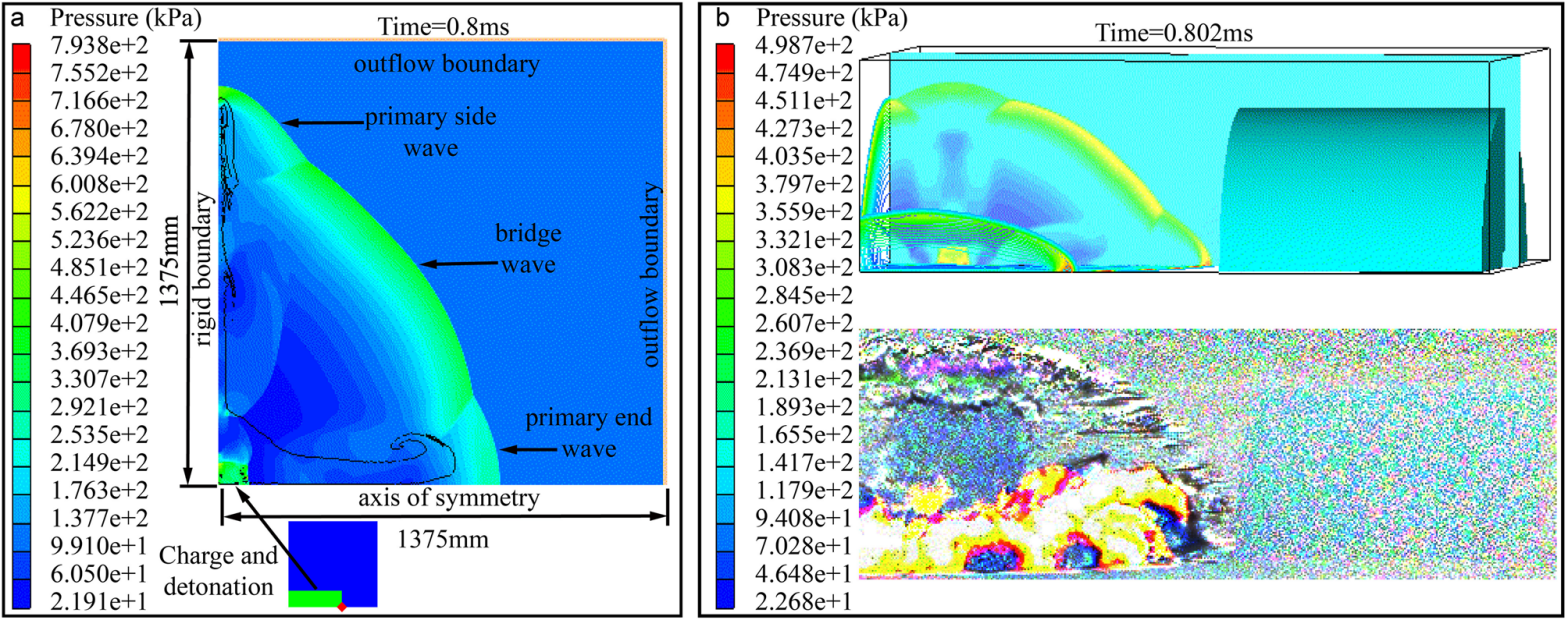
2D result mapping (adapted from Ref.^15^). (**a**) The 2D model and simulation results. (**b**) The simulation results of 3D model and test results.

Covering both the charge and the shell requires a relatively large air domain, and it is difficult to use a fine mesh in the 3D (three-dimensional) model considering computational efficiency. The 2D result mapping can be adopted in Case 1 owing to the axial direction of cylindrical charges being perpendicular to the ground (Fig. 9a,b), the material parameter, specific simulation methods, and verification can be found in ref.^15^.

**Figure 10:**
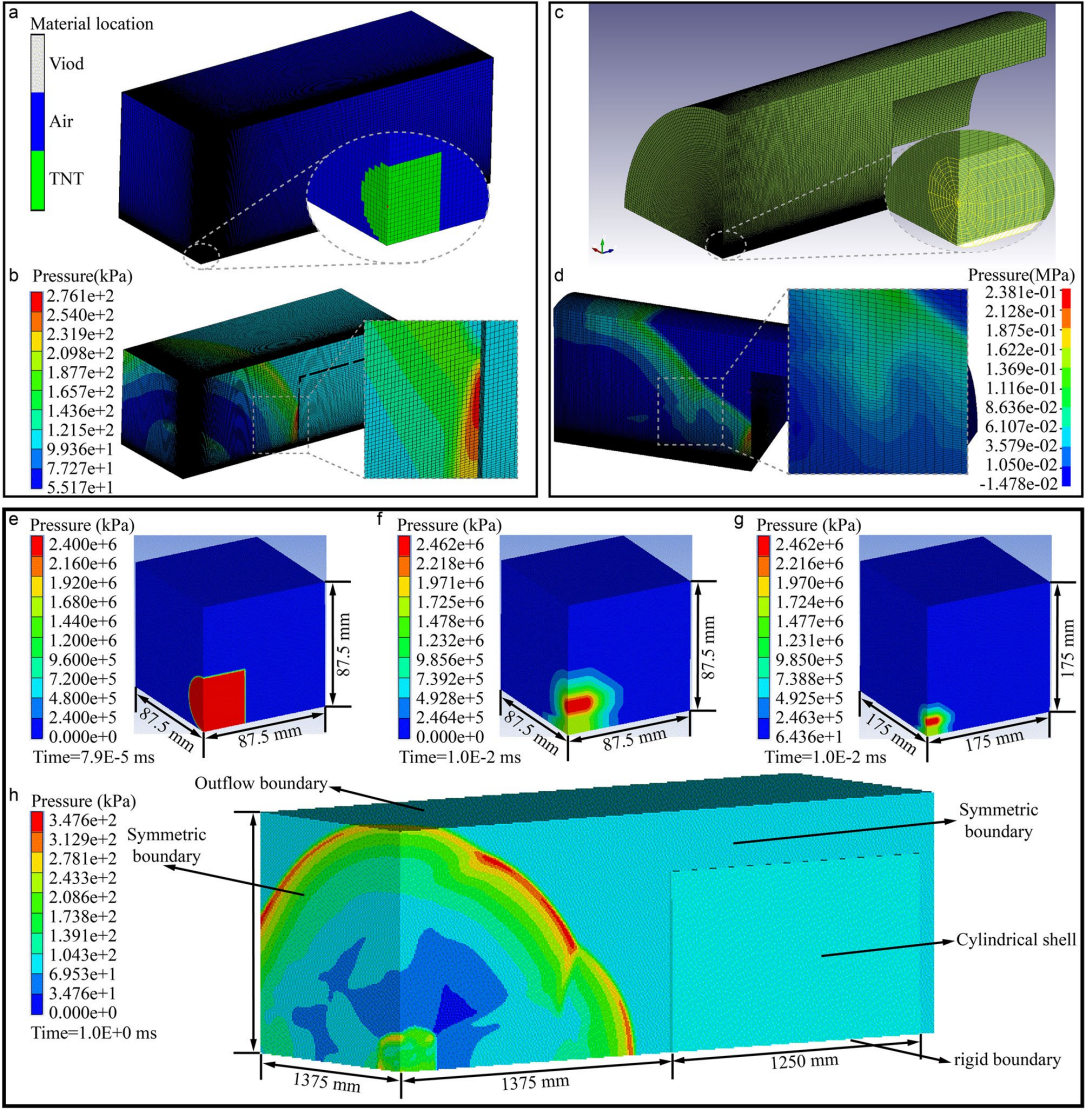
Gradient grid and block remapping. (**a,b**) Gradient grid model and simulation results in AUTODYN. (**c,d**) Gradient grid model and simulation results in LS-DYNA. (**e–h**) Block remapping process and simulation results in AUTODYN. (**e**) Initial pressure distribution. (**f**) Simulation results on 0.625 mm grids. (**g**) Block remapping to the model of 1.25 mm grids. (**h**) Final model after 5 consecutive block remaps.

The 2D result remapping affected by ground reflection can not be used for other cases except for Case 1, for Case 3, the gradient grid method is first adopted (Fig. 10a–d), that is, dense grids are used near charges and coarser grids are used at the low-pressure area, the strong discontinuity of secondary end waves cannot be simulated in AUTODYN (Fig. 10b), in LS-DYNA, relatively complex gradient grids can be easily divided, and the keyword *INITIAL_VOLUME_FRACTION_GEOMETRY is used to fill charges of various shapes, although secondary end waves are successfully simulated (Fig. 10d), the stratification of waves is fuzzy due to the coarse grid near cylindrical shells, and the corresponding peak overpressure is smaller than test results (Fig. 6c).

Since the block remapping function can only be used in the Euler-FCT solver, where TNT (charge) is directly converted to air pressure after fill (Fig. 10e), this automatically ignores the effect of detonation point on the detonation process, the block remapping in AUTODYN can be used to remap the simulation results of fine mesh (Fig. 10f) to the 3D model in larger air domain (Fig. 10g), this method has a good effect on the simulation of secondary end waves in Case 3, the stratification of the two waves is clear (Fig. 10h), the two peaks in overpressure-time histories are consistent with test results (Fig. 6c). The 3D block remapping has high accuracy for the blast loads of complex charges, but this method needs to adjust the air domain constantly, so the whole modeling process is complicated.

## Conclusion

The static images of the blast waves are better obtained by pixel subtraction, the position of detonators has a greater effect on detonation products in the near-field blasts. The difference between blast loads of the end face in different charge orientations is nearly double. When the axial direction of cylindrical charges points towards the cylindrical shell, the blast loads on the edge of cylindrical shells have a secondary peak, which the peak overpressure and maximum impulse are reduced by twice, the second peak is an extension wave formed by the bridge wave behind the end wave, not the second shock fronts captured in the camera. At the same time, the repeated test of Case 4 showed a huge difference, indicating that there is a sudden change in blast loads at a specific angle. For the real scenario of small-scale bomb attacks, in addition to charge equivalent and standoff distance considered in the scaled distance, more consideration should be given to the influence of charge shape, detonation configuration, and charge orientation. This paper provides a detailed simulation method which can simulate the blast loads of any charge orientations. The experimental and simulation methods can provide a reference for establishing a database of blast loads of typical building structures.

## Data Availability

The datasets used and/or analysed during the current study available from the corresponding author on reasonable request.
